# Activation profile of cGMP-dependent protein kinase Iα

**DOI:** 10.1186/2050-6511-14-S1-P79

**Published:** 2013-08-29

**Authors:** Sabine Wolter, Marina Golombek, Frank Schwede, Hans-Gottfried Genieser, Stefan Dove, Roland Seifert

**Affiliations:** 1 Institute of Pharmacology, Hannover Medical School, Hannover, Germany; 2 Biolog Life Science Institute, Bremen, Germany; 3 Department of Pharmaceutical/Medicinal Chemistry II, University of Regensburg, Regensburg, Germany

## Background

cGMP-dependent protein kinase (PKG) is a serine/threonine kinase which is potently activated by cGMP [1]. PKG is encoded by two genes, forming two different proteins, PKGI and PKGII. The two isoforms of PKGI, PKGIα and PKGIβ, differ in the N-terminal amino acid sequences. PKGI isozymes are homodimers with two identical subunits possessing a catalytic and a regulatory domain each. The regulatory domain contains two non-identical binding sites for cyclic nucleotides (cNMPs), i.e., a slowly exchanging and a rapidly exchanging site. The activation constant (K_a_) of PKGIα for cGMP is about 3-fold lower than the corresponding K_a_ of PKGIβ suggesting distinct physiological roles of the isoforms. In addition to cGMP, other cNMPs and also cNMP analogues activate or inhibit PKG [2-4]. While many investigations focussed on discrimination between the cNMP binding sites by employing cGMP and cAMP analogues, little is known about interaction of PKGIα with cCMP analogues or with *Rp*- and *Sp*- diastereomers of cCMP phosphorothioates.

As was shown by Desch et al. [5], the membrane-permeable cCMP analogue dibutyryl-cCMP (DB-cCMP) induces smooth muscle relaxation and activates PKGI in aortic tissue lysates. Therefore, we have studied 4-MB-cCMP, the resulting active metabolite after cleavage of DB-cCMP by esterases, and also corresponding substances from cAMP and cGMP, on purified PKGIα.

## Materials and methods

PKG kinase activity was measured *in-vitro* by a radiometric kinase assay in the presence of cGMP or different cNMP analogues. pEC_50_ values, K_a_, Hill slopes and E_max_ values were calculated using GraphPad Prism software. E_max_ values were related to E_max_ values of the activation of PKGIα by cGMP, which was set to 1.00.

## Results and discussion

Besides the known activator cGMP, many other cNMPs and cNMP analogues are activators of PKGIα, with distinct activation constants (pEC_50_), specific Hill slopes and different maximal effects (E_max_) (Table 1). The most potent and effective activator for PKGIα was cGMP. The active metabolite of DB-cGMP, 2-MB-cGMP was less potent and effective.

**Table 1:** pEC_50_, K_a_, Hill slopes and E_max_ for the activation of PKGIα by cNMPs.

cNMP	pEC_50_	K_a_ (µM)	Hill slope	E_max_
cGMP**	6.98 ± 0.04	0.11	1.71 ± 0.36	1.00 ± 0.04

2-MB-cGMP	5.84 ± 0.13	1.45	1.12 ± 0.34	0.66 ± 0.03

cAMP**	4.82 ± 0.11	15.13	1.28 ± 0.39	0.59 ± 0.05

6-MB-cAMP	4.67 ± 0.06	21.38	1.35 ± 0.19	0.81 ± 0.03

cCMP**	**4.58 ± 0.14**	**26.30**	**1.84 ± 0.53**	**0.55 ± 0.04**

**4-MB-cCMP**	**4.05 ± 0.10**	**89.13**	**1.10 ± 0.23**	**0.71 ± 0.06**

*Sp*-cAMPS	3.72 ± 0.61	190.55	1.38 ± 2.86	0.16 ± 0.01*

*Rp*-cAMPS	n.d.	n.d.	n.d.	n.d.

*Sp*-cCMPS	3.53 ± 0.97	295.12	1.11 ± 1.34	0.17 ± 0.02*

*Rp*-cCMPS	n.d.	n.d.	n.d.	n.d.

cIMP**	4.72 ± 0.04	19.05	1.54 ± 0.16	0.87 ± 0.02

cUMP**	4.15 ± 0.04	70.79	0.85 ± 0.21	0.72 ± 0.03

cXMP**	3.98 ± 0.04	104.71	2.06 ± 1.93	0.69 ± 0.03

*: value shows the maximum activation with 3 mM cNMP without saturation of the concentration/response curve

**: data from Wolter et al, Biochem Biophys Res Commun. 2011, **415**: 563-566.

n.d.: not determinable because of lack of saturation of the concentration/response curve.

cAMP and 6-MB-cAMP showed similar potency, but 6-MB-cAMP had a higher efficacy than cAMP. 4-MB-cCMP was a more effective activator than cCMP, but showed a reduced potency.

The cNMP analogues activated PKGIα in the order of potency cGMP > 2-MB-cGMP > cAMP > 6-MB-cAMP > cCMP > 4-MB-cCMP and in the order of efficacy cGMP > 6-MB-cAMP > 4-MB-cCMP > 2-MB-cGMP > cAMP > cCMP.

*Rp*-cAMPS and *Rp*-cCMPS did not activate PKGIα. The stable phosphorothioates *Sp*-cAMPS and *Sp*-cCMPS activated PKGIα only at high concentrations in the order of potency and efficacy cGMP > cAMP > cCMP >*Sp*-cAMPS ~ *Sp*-cCMPS.

Furthermore, we illustrate binding of cNMPs for PKG based on existing crystal structures and discuss current problems with respect to molecular modelling approaches. In conclusion, 4-MB-cCMP is a more effective PKG activator than cCMP and, therefore, a valuable tool for analysing the second messenger role of cCMP [6].
